# 17β-Estradiol Regulates the Sexually Dimorphic Expression of BDNF and TrkB Proteins in the Song System of Juvenile Zebra Finches

**DOI:** 10.1371/journal.pone.0043687

**Published:** 2012-08-31

**Authors:** Yu Ping Tang, Juli Wade

**Affiliations:** Neuroscience Program, Departments of Psychology and Zoology, Michigan State University, East Lansing, Michigan, United States of America; Utrecht University, The Netherlands

## Abstract

Mature brain derived neurotrophic factor (BDNF) plays critical roles in development of brain structure and function, including neurogenesis, axon growth, cell survival and processes associated with learning. Expression of this peptide is regulated by estradiol (E2). The zebra finch song system is sexually dimorphic – only males sing and the brain regions controlling song are larger and have more cells in males compared to females. Masculinization of this system is partially mediated by E2, and earlier work suggests that BDNF with its high affinity receptor TrkB may also influence this development. The present study evaluated expression of multiple forms of both BDNF and TrkB in the developing song system in juvenile males and females treated with E2 or a vehicle control. Using immunohistochemistry and Western blot analysis, BDNF was detected across the song nuclei of 25-day-old birds. Westerns allowed the pro- and mature forms of BDNF to be individually identified, and proBDNF to be quantified. Several statistically significant effects of sex existed in both the estimated total number of BDNF+ cells and relative concentration of proBDNF, varying across the regions and methodologies. E2 modulated BDNF expression, although the specific nature of the regulation depended on brain region, sex and the technique used. Similarly, TrkB (both truncated and full-length isoforms) was detected by Western blot in the song system of juveniles of both sexes, and expression was regulated by E2. In the context of earlier research on these molecules in the developing song system, this work provides a critical step in describing specific forms of BDNF and TrkB, and how they can be mediated by sex and E2. As individual isoforms of each can have opposing effects on mechanisms, such as cell survival, it will now be important to investigate in depth their specific functions in song system maturation.

## Introduction

Brain-derived neurotrophic factor (BDNF) is critical for diverse aspects of brain development and function, including cell survival, axon guidance, synaptic connectivity, dendritic arborization, long-term potentiation, and memory consolidation. The peptide is synthesized via precursors, prepro- then pro-BDNF, which is cleaved and secreted in the mature form. This secretion can occur in a regulated, activity dependent manner from either axons or dendrites (thus having anterograde or retrograde action), or via more passive, constitutive mechanisms [1], [2], [3].

BDNF binds to two types of receptors in the brain, with high-affinity to tyrosine kinase B (TrkB; [4], [5], [6]) and with low-affinity to the p75 receptor [7], [8]. All neurotrophins bind to the p75 receptor [9], thus its functions are not specific to BDNF. TrkB is more selective; it is the high affinity receptor for BDNF and neurotrophin-4 (NT-4). Isoforms of TrkB exist. The full-length form (TrkB-FL) contains a cytoplasmic domain that activates a variety of signaling cascades [10]. It is through this receptor that the vast majority of the enhancing effects on neuronal structure and function are elicited. However, an alternatively spliced variant (truncated; TrkB-T) lacks this intracellular portion and generally inhibits BDNF action (reviewed in [11]; see below).

Steroid hormones and BDNF interact. In particular, estradiol (E2) increases expression of BDNF mRNA and protein selectively *in vivo* and *in vitro* [12], [13], [14], [15]. mRNAs for estrogen receptors are co-expressed with BDNF and/or TrkB in a variety of forebrain regions in the developing rodent [15], [16], [17], [18]. While E2 does not appear to modulate TrkB expression in some situations (*e.g.*, developing male rat hippocampus [15]), E2 does increase TrkB protein in hypothalamic neuronal cultures from male rat brains, which is necessary for estrogenic effects on axon growth [19].

The song control system of zebra finches has long been an important model for investigating the effects of E2 on development of neural structure and function. Only males of this species sing, and most of the brain regions that control song learning and production are larger in males compared to females [20], [21]. Song control regions include the lateral magnocellular nucleus of the anterior nidopallium (LMAN) and Area X, which are critical to song development, and the HVC (used as a proper name) and robust nucleus of the arcopallium (RA), which are involved in the motor production of song. E2 treatment in female zebra finches during the first few weeks after hatching can masculinize song control nuclei (particularly HVC, RA and Area X) by increasing cell number and size, as well as the volume of those areas. Developmental treatment with E2 also enables females to sing in adulthood. However, the E2 alone cannot fully masculinize the song system in female zebra finches [20], [21], suggesting that other factors may be involved in the process of sexual differentiation. This notion is supported by studies that failed to prevent masculinization of song nuclei and the development of singing behavior by inhibiting the availability or action of estrogen in the early life of male zebra finches [22], [23], [24], [25], [26], [27], [28], [29], [30]. These studies also raise some questions about the role E2 might play in developing males. At the juvenile stages investigated, both plasma levels and the capacity for neural synthesis of the hormone are generally equivalent in the two sexes (reviewed in [20]).

One possibility is that E2 serves to increase BDNF protein, which subsequently contributes to the masculinization process. Previous work has indicated that E2 treatment of juvenile males and females results in an increase of BDNF mRNA in HVC. Moreover, inhibition of estrogen synthesis blocks an increase of BDNF mRNA expression seen in males in this region between post-hatching days 25 and 35 [31].

Sex chromosome genes may also be strong possibilities for facilitating masculine development [32]. Male birds are ZZ, and females ZW. Because dosage compensation in birds is limited, the expression of Z-genes is greater in males compared to females [33], including within specific song control nuclei [34], [35], [36], [37], [38], [39], [40]. TrkB is on the Z-chromosome, and its mRNA exhibits higher expression in the song system of developing males [41]. TrkB protein was also detected in the RA of males at 15–20 days of age [42], and across the song system at later developmental stages [43]. Up-regulation of this receptor could provide a mechanism for increased BDNF action in song system masculinization.

Thus, BDNF may facilitate masculinization of zebra finches via two mechanisms. E2 may increase availability of the protein (which might occur in both sexes to some extent) and higher expression of TrkB in males may increase its ability to act. To further elucidate mechanisms regulating masculinization, the effect of E2 on BDNF was investigated in juvenile male and female zebra finches. We evaluated protein, both the number of cells expressing BDNF and its relative concentration, across the forebrain song control regions. Because potential effects of E2 on TrkB have not been reported, we also investigated this protein in song nuclei using Western blot analyses to distinguish between the full length and truncated forms of TrkB.

## Materials and Methods

### Animals

Zebra finches were raised in walk-in colonies, containing approximately 7 adult males and females and their offspring. The birds were exposed to a 12∶12 light∶dark cycle. Finch seed and water were continuously available, and a mixture of bread and hard-boiled chicken eggs, as well as spinach and orange, were provided once a week. Nests boxes were checked daily; the day a hatchling was found was post-hatching day 1. All procedures were conducted in accordance with NIH guidelines and approved by the Michigan State University IACUC.

### Hormone treatment and tissue collection

Males and females each received a subcutaneous implant of either 17β-estradiol (Steraloids, Welton, NH) or a blank pellet (n = 6 per sex per treatment) on post-hatching day 3 (as in [37]). Hormone implants were produced using a 1∶5 mixture E2 and silicone sealant (Dow Corning, Midland, MI) that was expelled in a line through a 3-cc syringe onto wax paper and dried overnight. The mixture was cut into 1 mm lengths and then quartered, so that each implant contained approximately 100 µg of E2. Control blank pellets (BL) were produced identically, except that they did not contain the hormone. Each animal was rapidly decapitated at 25 days of age. Brains were separated into two hemispheres, frozen in cold methyl-butane and stored at −80°C. The left and right sides were randomly selected for use in Western blot analyses and immunohistochemsitry. Sex was determined at this time by visual examination of the gonads.

**Figure 1:**
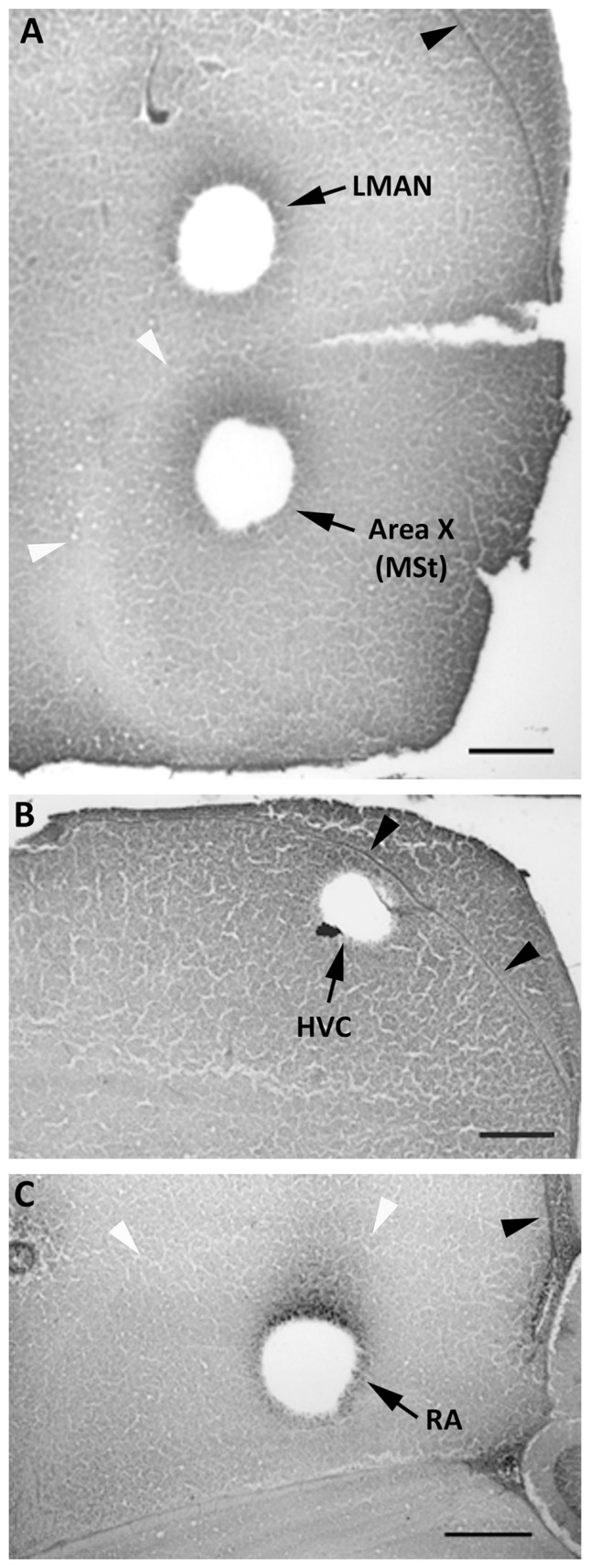
Photographs demonstrating the punches of song control nuclei collected for Western blot analyses. All images are from females, and show typical collections from LMAN and the MSt (A), HVC (B), and RA (C). Black arrowheads point to the lateral ventricles. White arrowheads indicate the pallial-subpallial lamina (A) and dorsal arcopallial lamina (C). Scale bars = 500 µm.

For Western blots, one hemisphere of each brain was sectioned at 50 µm, and punches (ranged from 9 to 25 depending on song nucleus size) were collected individually with a stainless steel cannula (0.5 mm diameter; Stoelting Co., Wood Dale, IL) from each section in which HVC, RA, LMAN, and Area X could be identified (Figure 1). These regions are readily identified based on a variety of visual characteristics, including surrounding landmarks such as fiber tracts and the lateral ventricles, and differences in color and consistency compared to surrounding tissue. The first three areas were collected from both sexes, but as Area X cannot be detected in control females, a comparable portion of the medial striatum (MSt) was obtained from control females based on landmarks. Tissue was expelled into 100 µl of RIPA lysis buffer (sc-24948; Santa Cruz Biotechnology, Santa Cruz, CA) and stored at −20°C until protein extraction. The remainder of each section was Nissl-stained to confirm accuracy of the punches (Figure 1).

For immunohistochemistry, the remaining hemisphere from each bird was cut frozen at 20 µm into six alternate series of sections. Slides were stored at −80°C with desiccant until processing.

### BDNF and TrkB Western Blot Analyses

**Figure 2:**
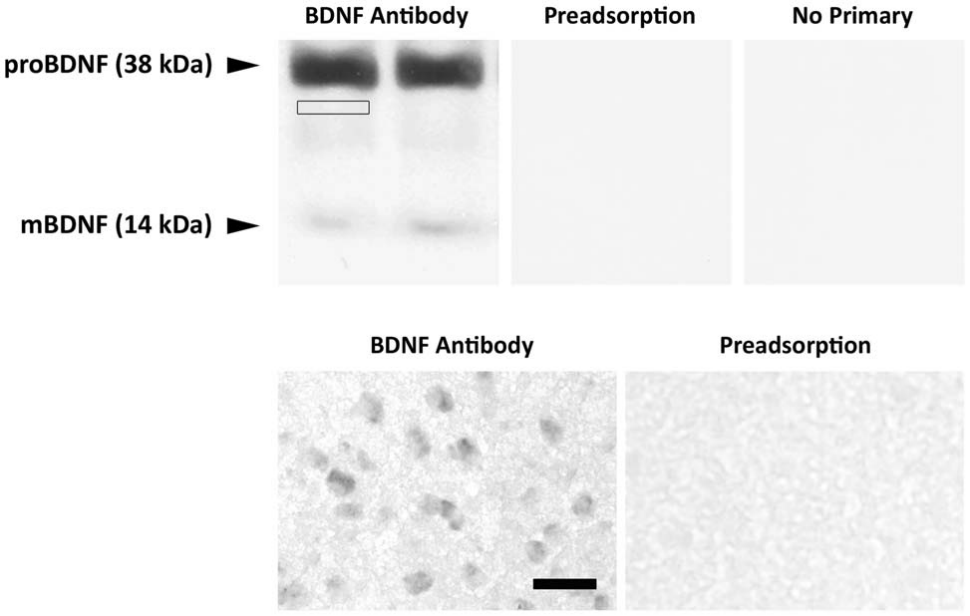
BDNF antibody specificity. Top: Western blot analyses used total protein (30 µg) extracted from the whole telencephalon of two 25-day-old male zebra finches. The left image shows two bands (38 kDa proBDNF and 14 kDa mature mBDNF) recognized by BDNF primary antibody. The small box immediately below the proBDNF band indicates an example of where the background optical density would be quantified. The middle and right images document the absence of these bands on separate blots preadsorbed with 20-fold excess of the antigen and with the primary antibody omitted, respectively. Bottom: Immunohistochemistry showing distinct BDNF+ cells in RA with the primary antibody and a complete absence of labeling in neighboring section exposed to the antibody following preadsorption with 10-fold excess peptide. Scale bar = 30 µm for both photographs.

The specificity of BDNF antibody was examined using Western blot analyses with 30 µg total protein from the whole telencephalon of two juvenile male zebra finches. Two bands, representing the 38 KDa proBDNF and 14 KDa mature BDNF, were recognized by the antibody (see below). Labeling on both Western blots and in tissue sections used for immunohistochemistry was completely eliminated when the primary antibody was preadsorbed with the peptide against which it was raised, as well as when the BDNF primary antibody was omitted (Figure 2). Specificity of the TrkB antibody was previously verified [43].

Experimental analyses were completed on protein extracted from the samples of HVC, RA, Area X (or an equivalent portion of the MSt in control females), and LMAN of each individual using RIPA lysis buffer per manufacturer's instructions. Concentrations were quantified with the Bradford method (Bio-Rad; Hercules, CA). Samples (8 µg of total protein, which was the maximum available for some individuals) from each brain region were run on three precast gels (Any kD Mini-PROTEAN TGX; Bio-Rad; Hercules, CA) with each gel containing 2 samples from each group and a ladder for size determination (Precision Plus Dual Color Standard; Bio-Rad). The protein was then transferred to PVDF membranes at 4°C. Membranes were simultaneously treated with the SuperSignal Western Blot Enhancer kit (Thermo Scientific; Rockford, IL) according to manufacturer's instructions, and blocked with SuperBlock Blocking Buffer (Thermo Scientific; Rockford, IL) for 60 minutes at room temperature to eliminate non-specific binding. Membranes were then incubated with the BDNF (N-20) primary antibody (1 µg/ml; sc-546; Santa Cruz Biotechnology, Santa Cruz, CA) in primary antibody diluent from SuperSignal Western Blot Enhancer kit overnight at 4°C. Horseradish peroxidase-conjugated goat anti-rabbit secondary antibody (1∶5,000; Cell Signaling, Danvers, MA) was applied to the membranes at room temperature for 1 hour. Immunoreactivity was detected by chemiluminescence (SuperSignal West Pico, Thermo Scientific; Rockford, IL) followed by exposure to HyBlot CL autoradiography film (Denville Scientific Inc.; Metuchen, NJ).

The membranes were stripped in Restore Plus Western Blot Stripping Buffer (Pierce; Rockford, IL) per manufacturer's instructions, washed in 1X PBS-Tween 20, and re-probed for actin as a loading control (0.5 µg/ml, sc-1615; Santa Cruz Biotechnology; Santa Cruz, CA) overnight at 4°C. They were then incubated with HRP-conjugated donkey anti-goat secondary antibody (1 µg/30 ml, sc-2020; Santa Cruz Biotechnology, Santa Cruz, CA) for 1 hour at room temperature, and the reaction product was visualized as above. Finally, each membrane was stripped again as described above and re-probed with the TrkB primary antibody (1 µg/ml, sc-12; Santa Cruz Biotechnology; Santa Cruz, CA) followed by goat anti-rabbit-HRP secondary antibody (1∶5,000; Cell Signaling, Danvers, MA).

The 38 KDa proBDNF band was expressed in all the samples. However, the 14 kDa mBDNF band was only detectable in some (Table 1), even after prolonged exposure to film (1 hour for HVC; 2–2.5 hours for LMAN and RA; Area X/MSt never produced a clear signal). The long exposure required to detect mBDNF bands resulted in high background, so the relative optical density could not be quantified. Therefore, only the ratio of proBDNF to actin from each sample was analyzed for each brain region mentioned above. While the truncated form of TrkB (TrkB-T, 95 KDa) was detected in each brain region examined, the full length TrkB (TrkB-FL, 145 KDa) was consistently observed in HVC only. Therefore, the ratio of TrkB-T/actin was analyzed in all song nuclei and the ratio of TrkB-FL/actin was quantified only in HVC.

**Table 1:** Number of animals from each group in which a distinct 14 kDa band representing the mature form of BDNF was detected in each song nucleus using Western blot analysis.

	LMAN	Area X (MSt)	HVC	RA
**Male Control**	2	0	5	6
**Male E2**	3	0	6	5
**Female Control**	2	0	3	5
**Female E2**	1	0	4	5

Sample size was 6 in each case.

The mean optical density for each band of interest was quantified using Image J (NIH). A value was also obtained for an immediately adjacent region of the same size (Figure 2), which was subtracted to control for background. The ratio of proBDNF or TrkB to actin was then calculated and analyzed by two-way-ANOVA (sex x treatment) in each brain region, followed by pairwise planned comparisons as appropriate. A few samples could not be included in statistical analyses due to a poor film image; final sample sizes are included in the figures.

### BDNF Immunohistochemistry

One set of slides from each animal was warmed to room temperature, rinsed in 0.1 M phosphate-buffered saline (PBS), fixed in 4% paraformaldehyde for 15 minutes, and washed 3 times in PBS. Slides were exposed to 0.9% H_2_O_2_/methanol for 30 minutes and incubated for 30 minutes in 3% normal goat serum in PBS with 0.3% Triton X-100. The tissue was then incubated in 0.1 M PBS containing 0.3% Triton X-100, 3% NGS and BDNF primary antibody (0.5 µg/ml; same as used for western blot) overnight at 4°C. A biotin-conjugated goat anti-rabbit secondary antibody (1 µg/ml; Vector Labs, Burlingame, CA) was then applied for 1.5 hours at room temperature, followed by treatment with *Elite* ABC reagents and diaminobenzidine (DAB) with 0.0024% hydrogen peroxide to produce a brown reaction product. Slides were then rinsed in PBS to be sure the reaction was terminated. An adjacent set of slides from each animal was stained with Cresyl violet to localize the song control nuclei. Slides were coverslipped with DPX (Fluka, St. Louis, MO) after dehydration in a graded series of ethanols.

### Stereological Analyses

LMAN, HVC and RA were analyzed in both males and females. However, Area X cannot be detected with a Nissl stain in female zebra finches, and borders also could not be distinguished with BDNF labeling. Therefore, labeled cells in this region were only quantified in males and E2-treated females. As a control, we analyzed BDNF expression in nucleus rotundus (RT), a sexually monomorphic thalamic nucleus [44], [45] to determine whether the effects of E2 were specific to song nuclei. Regions of interest from each animal were analyzed under brightfield illumination using StereoInvestigator software (Microbrightfield Inc., Williston, VT) by an individual blind to sex and age of the animals. The border of each song nucleus was defined by tracing its edge throughout its rostrocaudal extent. All cells exhibiting neuronal morphology and clear reaction product for BDNF were manually counted in regions selected by the Optical Fractionator function [39]. Due to tissue quality, data for all brain regions could not be obtained from every individual. Final sample sizes are indicated in the figures.

Within HVC, RA, LMAN and RT, the estimated total number of BDNF+ cells was analyzed by 2-way ANOVA. Main effects of sex and treatment, as well as potential interactions between the variables were assessed. Planned, pairwise comparisons were conducted when sex x treatment interactions existed. Because the region is not detectable in control females, in Area X the estimated total number of BDNF+ cells was analyzed by one-way ANOVA among control males, E2 treated males and E2 treated females.

## Results

### proBDNF western blot analyses

#### LMAN

**Figure 3:**
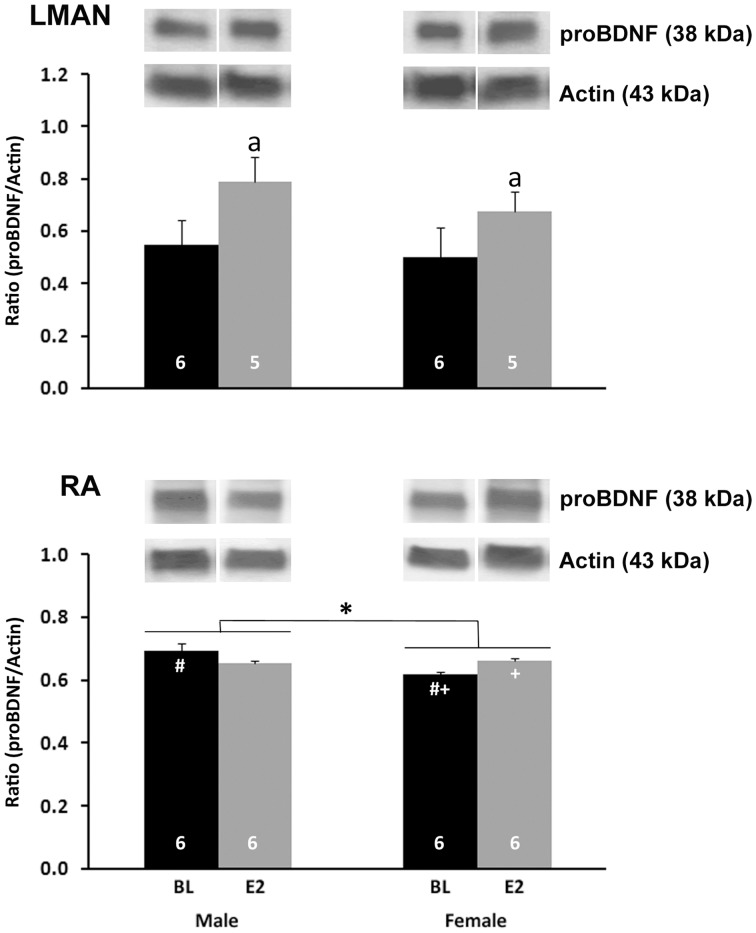
proBDNF protein in song control nuclei. Data and representative images are from the punches of LMAN (top) and RA (bottom). The ratio of proBDNF/actin was calculated from mean optical densities of bands in each individual. Values represent means+one standard error. Symbols indicate significant differences as follows: a = main effect of treatment; * = main effect of sex; # = control males greater than control females; + = E2 females greater than control females. Sample sizes are indicated at the bottom of each bar. Representative bands for each protein from the LMAN and RA of each group are shown above the histograms.

A significant main effect of treatment was detected (F_1,18_ = 4.610, P = 0.046), with an increase in pro-BDNF/actin ratio in E2-treated compared to control animals (Figure 3, top). Neither a main effect of sex nor an interaction between sex and treatment was found (both F_1,18_<0.686, P>0.418).

#### RA

A significant main effect of sex (F_1,20_ = 5.816, P = 0.026) and an interaction between sex and treatment (F_1,20_ = 9.422, P = 0.006) were detected (Figure 3, bottom). Control males had a higher proBDNF/actin ratio than control females (t_10_ = 9.647, P = 0.011), but the values did not differ between the sexes in birds treated with E2 (t_10_ = 0.489, p = 0.500). E2 significantly increased the proBDNF/actin ratio in females only (t_10_ = 15.327, P = 0.003; males: t_10_ = 2.555, p = 0.141). No main effect of treatment was detected (F_1,20_ = 0.024, p = 0.877).

#### HVC and Area X (MSt)

No effects of sex, treatment or interaction were observed in these remaining areas (all F<3.494, P>0.077; data not shown).

### TrKB western blot analyses

#### LMAN

**Figure 4:**
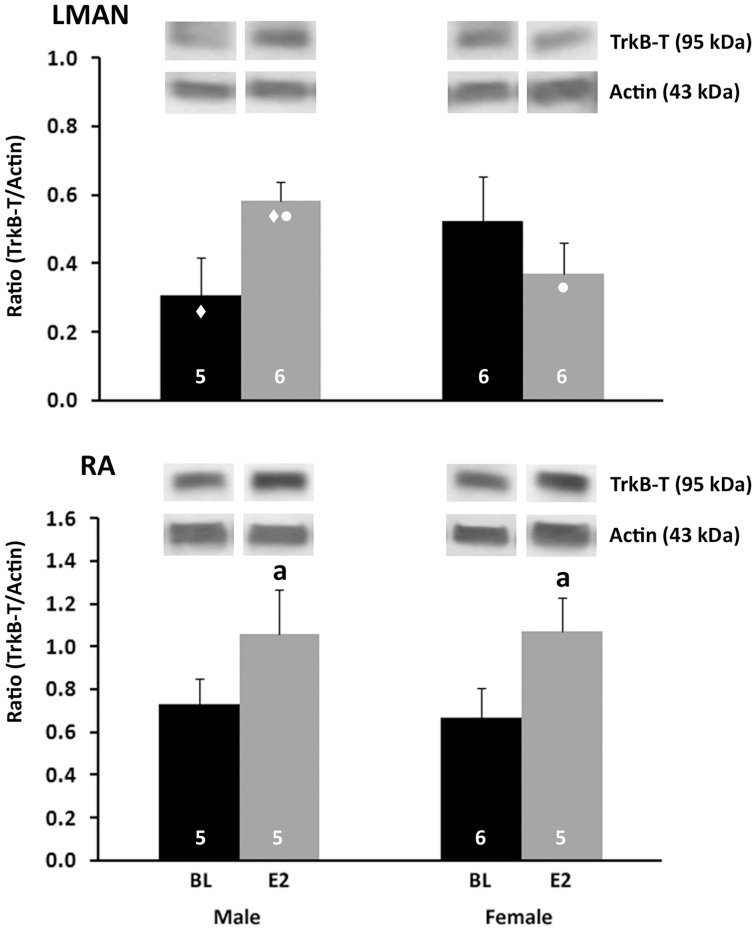
Quantification of TrkB-T protein. Data and representative bands from Western blots analyses of LMAN (top) and RA (bottom) punches are depicted. The ratio of TrkB-T/actin was calculated from mean optical densities of bands representing these proteins in each individual. Values indicate means+one standard error. Symbols represent trends as follows: ♦ = TrkB-T is greater in E2-treated than control males, p = 0.053; • = In E2-treated birds, TrkB-T is decreased in females compared with males; p = 0.052. The letter ‘a’ indicates a significant main effect of treatment. Sample sizes are indicated within the bars for each group. Representative bands for each protein from the LMAN and RA of each group are shown above the histograms.

No main effects of sex (F_1,19_<0.001, P = 0.991) or treatment (F_1,19_ = 0.345, P = 0.564) were found on the TrkB-T/actin ratio. A significant interaction between sex and treatment was detected (F_1,19_ = 4.657, P = 0.044), which suggested that E2 might have different effects in males and females. However, pairwise comparisons indicated only trends for significant differences (Figure 4, top).

#### RA

A significant main effect of treatment was detected in the ratio of TrkB-T/actin (F_1,17_ = 6.308, P = 0.022). This value was increased in E2-treated birds compared to control animals (Figure 4, bottom). Neither a main effect of sex nor an interaction between sex and treatment was detected (both F_1,17_<0.074, P>0.789).

#### HVC

**Figure 5:**
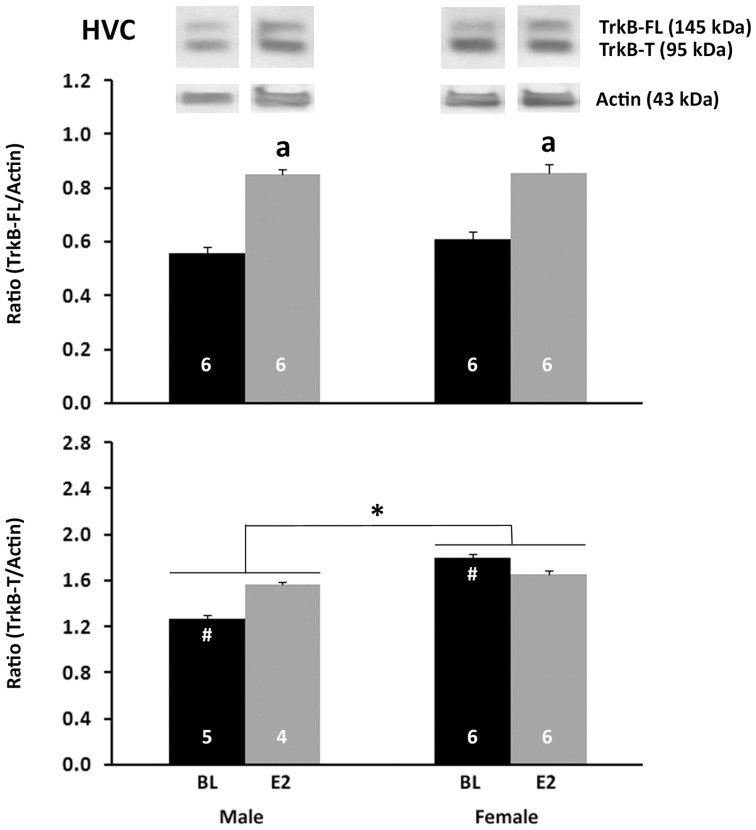
Western blot analyses of TrkB proteins in punches of HVC. The full length receptor (TrkB-FL) is shown on the top, and the truncated form (TrkB-T) on the bottom. The ratio of each of these proteins relative to actin was calculated from mean optical densities for each individual. Values indicate means+one standard error. Symbols indicate significant effects as follows: a = main effect of treatment; * = main effect of sex; # = Control female>control male. Sample sizes are indicated in the bars. Representative bands for each protein from are shown above the histograms.

For the ratio of TrkB-FL/actin (Figure 5, top), a main effect of treatment was detected with E2 increasing the value compared to the control (F_1,20_ = 6.864, P = 0.019). There was no main effect of sex (F_1,20_ = 0.075, P = 0.788) and no interaction between sex and treatment (F_1,20_ = 0.050, P = 0.825). For the ratio of TrkB-T/actin (Figure 5, bottom), a significant main effect of sex was detected, such that it was greater in females than males (F_1,17_ = 8.241, P = 0.011). An interaction between sex and treatment was also detected for TrkB-T/actin (F_1,17_ = 4.455, P = 0.050). Pairwise comparisons revealed a higher level in control females than control males (t_9_ = 10.537, P = 0.010).

#### Area X (MSt)

No significant effects of sex, treatment or interaction between sex and treatment were found in TrkB-T/actin ratio (all F_1,19_<0.923, P>0.349; data not shown).

### Estimated total number of cells labeled with BDNF Antibody

#### LMAN

**Figure 6:**
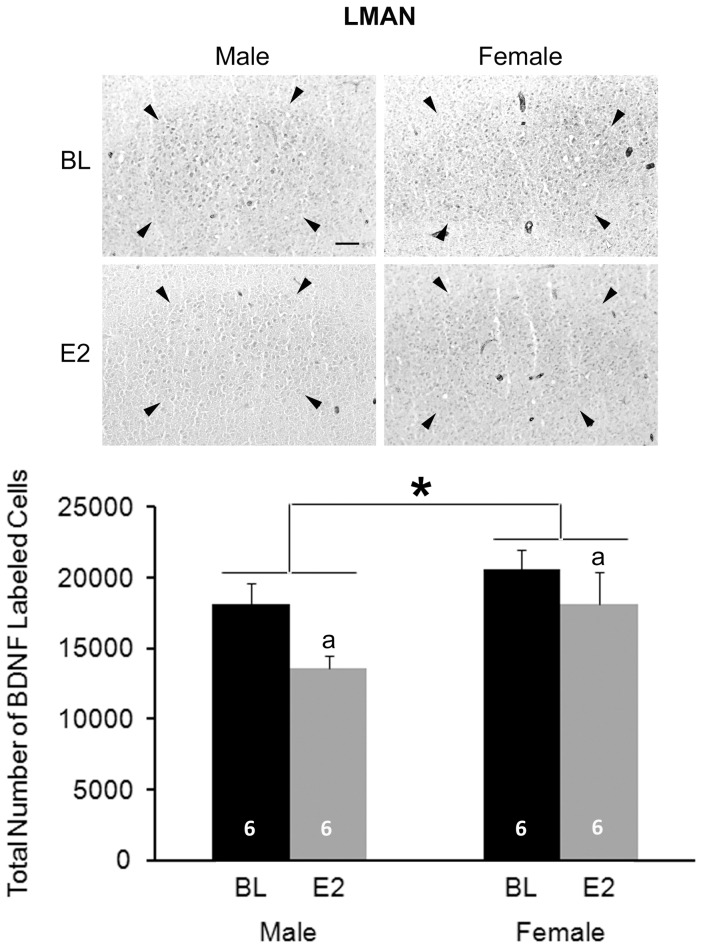
Immunohistochemistry for BDNF in LMAN. The top panels are photographs from a representative individual of each group. Arrow heads indicate the border of LMAN. Scale bar for all images (shown in top left photo) = 100 µm. On the bottom, the histogram shows means+one standard error for the estimated total number of BDNF+ cells for each group (bottom). * = significant main effect of sex; a = significant main effect of treatment. Sample sizes are indicated within each bar.

A significant main effect of sex was detected (F_1,20_ = 4.807, P = 0.040), with females having more BDNF+ cells than males. Main effect of treatment also existed (F_1,20_ = 4.743, P = 0.042); E2 decreased the number of BDNF+ cells compared to the controls (Figure 6). There was no interaction between sex and treatment (F_1,20_ = 0.457, P = 0.507).

#### RA

**Figure 7:**
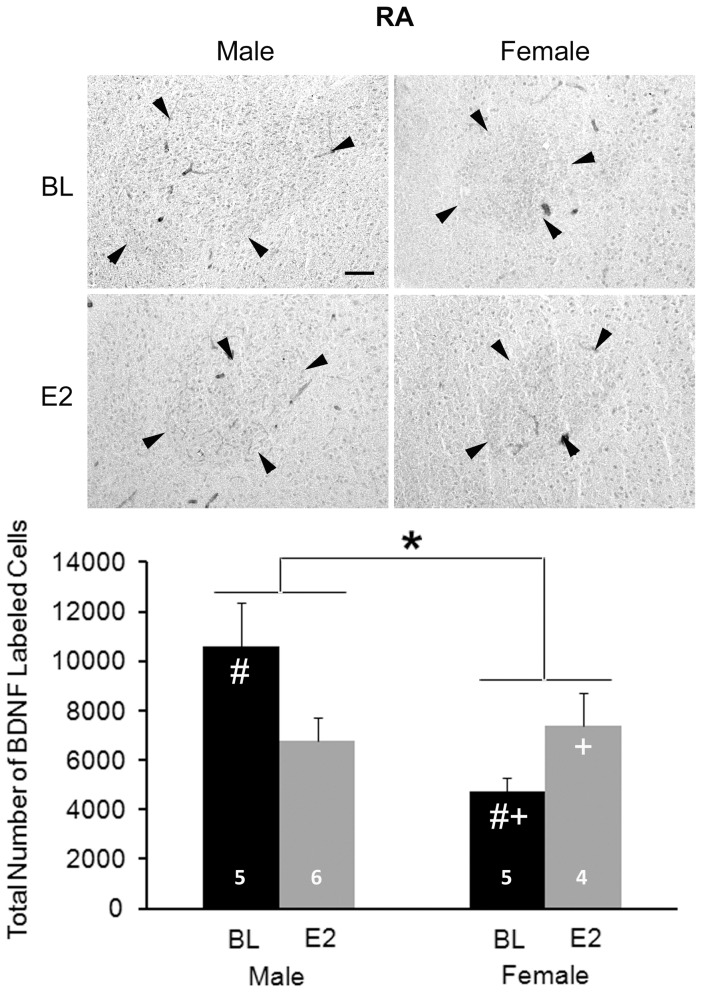
Immunohistochemistry of BDNF in RA. The top panels are photographs from a representative individual of each group. Arrow heads indicate the border of RA. Scale bar for all images (shown in top left photo) = 100 µm. On the bottom, the histogram shows means+one standard error for the estimated total number of BDNF+ cells for each group (bottom). * = significant main effect of sex; # = sex difference within control birds; + = significant effect of E2 within females. Sample sizes are indicated in the bars.

A significant main effect of sex (F_1,16_ = 5.544, P = 0.032), but not treatment (F_1,16_ = 0.260, P = 0.617), was detected. An interaction between sex and treatment was also seen (F_1,16_ = 8.534, P = 0.010; Figure 7). Control males had more BDNF+ cells than control females (t_8_ = 3.503, P = 0.008). In females, E2 increased the total number of BDNF+ cells (t_7_ = 2.569, P = 0.037). In males, E2 treatment resulted in 36% decrease of the total number of BDNF+ cells, but this effect was not statistically significant (t_9_ = 2.114, P = 0.064). This value was equivalent in E2-treated males and females (t_8_ = 0.432, P = 0.667).

#### HVC

**Figure 8:**
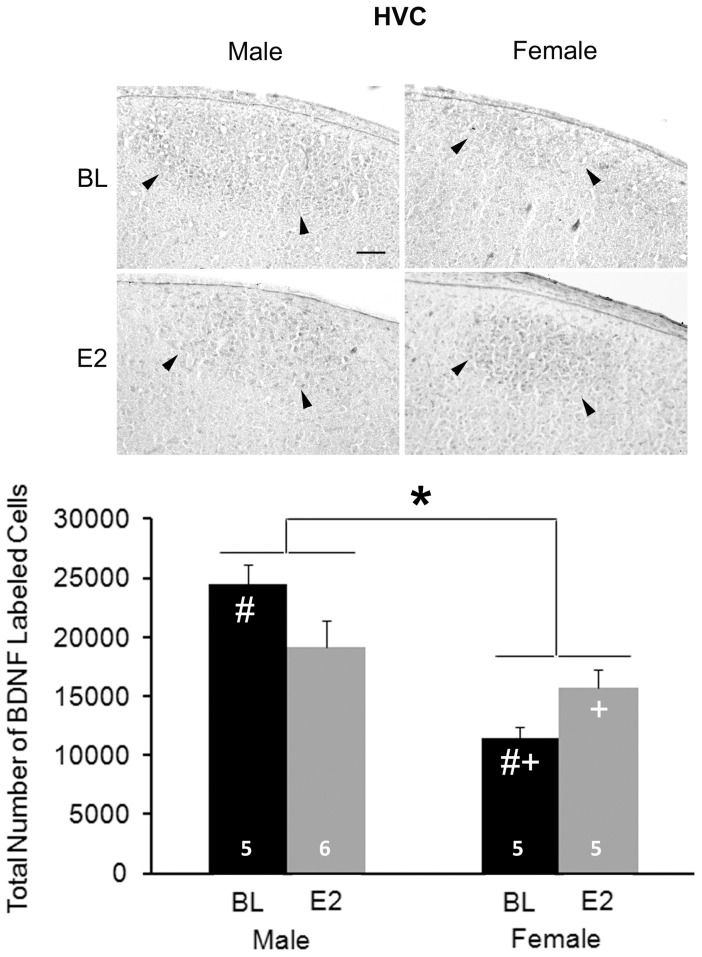
Immunohistochemistry of BDNF in HVC. The top panels are photographs from a representative individual of each group. Arrowheads indicate the ventral border of HVC. Scale bar for all images (shown in top left photo) = 100 µm. On the bottom, the histogram indicates means+one standard error for the estimated total number of BDNF+ cells (bottom). * = significant main effect of sex; # = sex difference within control animals; + = effect of estradiol within females. Sample sizes are indicated within the bars.

Overall, significantly more BDNF+ cells were detected in males than females (main effect of sex: F_1, 17_ = 22.489, P<0.001; Figure 8). A significant interaction between sex and treatment was also observed (F_1,17_ = 7.664, P = 0.013), but there was no main effect of treatment (F_1,17_ = 0.096, P = 0.761). Among control animals, males had a greater number of BDNF+ cells than females (t_8_ = 7.337, P<0.001). E2 significantly increased the number of BDNF+ cells in females (t_8_ = 2.646, P = 0.029), but had no effect in males (t_9_ = 1.804, P = 0.105). No difference between E2-treated males and females was detected (t_9_ = 1.188, P = 0.265).

#### Area X

The estimated total number of BDNF+ cells was equivalent across the three groups (F_2,15_ = 1.548, P = 0.245; data not shown).

#### RT

**Figure 9:**
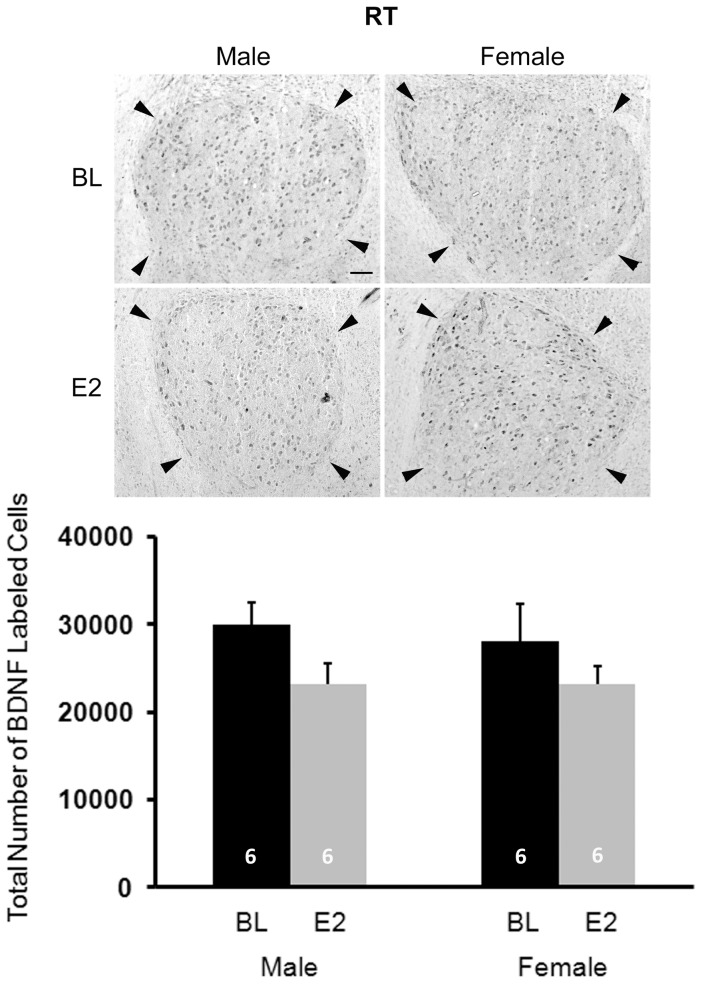
Immunohistochemical labeling of BDNF in the control nucleus rotundus (RT). The top panels are photographs from a representative individual of each group. Arrowheads depict the border of this brain region. Scale bar for all images (shown in top left photo) = 100 µm. On the bottom, the histogram indicates means+one standard error for the estimated total number of BDNF+ cells (bottom). No statistically significant effects of sex or treatment were detected, and no interaction between these variables existed.

No main effects of sex (F_1,20_ = 0.101, P = 0.754) or treatment (F_1,20_ = 3.798, P = 0.065) were detected, and no interaction between sex and treatment (F_1,20_ = 0.094, P = 0.763) existed (Figure 9).

## Discussion

### Summary

A number of effects of both sex and E2 were detected for BDNF. In both the HVC and RA, males had more cells expressing this protein, and E2 masculinized this characteristic in females. The results were opposite in LMAN, with more BDNF+ cells detected in females and E2 decreasing this value (in both sexes). As with other markers [20], [21], [39], [46], Area X could not be detected in control females in the present study. However, E2 induced a visible Area X defined by BDNF labeling.

Mature BDNF was consistently detected by Western blot, particularly in HVC and RA, but it could not be quantified due to limited protein availability; it was difficult to obtain sufficient signal with low enough background. However, proBDNF could be readily measured, and the patterns differed from those on BDNF+ cell number. Relative proBDNF concentration in RA was greater in males than females and was increased by E2 in females. This up-regulation by E2 was also seen in LMAN.

The present results provide novel information regarding BDNF on at least two levels. First, the HVC, RA and LMAN of unmanipulated, 25-day-old animals of both sexes express BDNF, as does the Area X of control males. Second, the availability of the protein can be modulated by E2, although the specific nature of the regulation depends on brain region, sex and form of the protein. The difference in the pattern of results between the Western blot analysis and immunohistochemistry suggests that at least some of what was quantified stereologically was mature BDNF. However, it is also possible that estimation of numbers of cells expressing this (or any) protein does not directly parallel its local concentration.

Like BDNF, the relative concentration of TrkB exhibited different patterns among the song nuclei. TrkB-T was significantly higher in control females than control males only in HVC. E2 had no effect in this region but increased the receptor in the RA of females. In contrast, no effect of sex was detected on the full-length form of this receptor (TrkB-FL, which was only detected consistently in HVC), and E2 significantly increased its expression in both males and females. The full-length vs. truncated forms of the TrkB receptor had not been distinguished in songbirds. The current data indicate that at least some of what has been detected in juveniles by immunohistochemistry across HVC, RA, LMAN and Area X is the truncated form. The data also indicate that expression of both isoforms can be increased by E2, depending on brain region.

### Functions of BDNF and TrkB isoforms

Mature BDNF positively regulates a range of effects on the central nervous system. These include increasing the proliferation, migration, survival and differentiation of neurons, as well as increasing synaptic plasticity. These actions occur via the full length TrkB receptor [47], [48], [49]. In contrast, proBDNF itself can be released either by cultured neurons *in vitro* [50], [51], [52] or by central neurons *in vivo* [53], suggesting it may have specific functions in the brain. ProBDNF has a variety of negative functions, including promoting cell death, decreasing dendritic spine density, inhibiting neuronal migration, and attenuating synaptic transmission; these effects occur via the p75 pan neurotrophin receptor [52], [53], [54], [55].

Similar to the pro- and mature forms of BDNF, the full-length and truncated forms of the TrkB receptors have largely opposite effects. The truncated form can bind and internalize/sequester BDNF, but does not undergo autophosphorylation or function as a tyrosine kinase receptor. TrkB receptors dimerize; full-length homodimers mediate the neurotrophic effects of BNDF. Heterodimers of full-length and truncated receptors have dominant-negative functions that inhibit signaling. Thus, the truncated form of the TrkB receptor generally serves to inhibit BDNF activity. However, independent of this ligand, homodimers of TrkB-T can induce some neurite outgrowth via mechanisms that are not well understood [11].

### BDNF and TrkB in the Song System

A variety of supportive roles of BDNF have been documented in songbirds. Infusion of this neurotrophin into RA during development prevents cell death following removal of presynaptic input by lesioning LMAN [42]. Injection into the adult zebra finch RA introduces song plasticity, suggesting that BDNF regulates variability in a manner critical to learning [56]. In adult canaries, which unlike zebra finches exhibit seasonal changes in song and morphology of song control regions, TrkB is present in the HVC of both sexes, and BDNF protein is in the HVC of males only, where it is involved in the regulation of neuronal replacement [57]. In male canaries, HVC BDNF mRNA is up-regulated by singing, and in parallel, the survival of new neurons is increased in singing birds [58]. Similar results are seen in white-crowned sparrows [59]; BDNF mRNA is increased in HVC by the long days typical of spring breeding conditions. Data from infusing and inhibiting BDNF in RA indicate its importance for seasonal plasticity of the song system in this species as well. While more work on BDNF function has been done in adulthood than development, collectively the data suggest the potential for BDNF acting at TrkB receptors to regulate a variety of aspects of structure and/or function of the song system.

Several studies have localized BDNF and TrkB in the developing song system. Unfortunately, a number of inconsistencies exist across the results, perhaps in part due to differences in methodology. BDNF mRNA has been detected in the HVC of males, but not females, at 30–35 days post-hatching. This expression was increased by E2. In contrast, BDNF mRNA was not detected in the RA of juveniles of either sex, and levels in LMAN were reported as being very low, but perhaps increased compared to surrounding tissue; this labeling was not in RA-projected LMAN cells [31]. Cell bodies containing BDNF protein were detected in the LMAN but not RA of 15–20 day old males; fibers were reported in RA of these birds [42]. Akutagawa and Konishi [60] reported BDNF protein in the HVC of males at day 20 and RA at day 45. BDNF emerged in the LMAN and Area X of males between days 45 and 65. This study reported very little BDNF immunoreactivity in the song system of adult males. In contrast, Johnson et al. [61] suggest that the labeling with this antibody in RA is due to a technical artifact and that BDNF in HVC is comparable to surrounding telencephalic tissue and equivalent in juvenile and adult birds.

One antibody to the extracellular domain of TrkB indicated cell bodies, neuropil and fibers in males at d15–20 in RA [42]. Another antibody, the one used in the present paper which also recognizes both isoforms, revealed labeling in somata and neuropil that appeared to define the HVC and RA in males from days 30–60, and in females on days 45 and 60. Scattered cells were also detected in LMAN. However, consistent labeling was not detected in the song system before these ages [43]. TrkB mRNA has been documented in the RA, HVC, and LMAN of juveniles in both sexes [31]. It also is expressed higher levels in the forebrain of males compared to females in the first week after hatching. Within HVC specifically, the mRNA expression is higher in males compared to females as early as 6 days after hatching [41].

### Synthesis of Existing Data

In the present study, relative proBDNF levels within HVC were unaffected by sex and E2 treatment, as determined by Western blot analysis. However, more cells expressing BDNF protein were detected in males than females, and E2 increased this measure in females only. The relationship between proBDNF concentration in protein homogenized from much of a song nucleus and the number of cells in which multiple forms of BDNF may be detected in the entire region is not completely clear. However, the divergence in the data suggest that one interpretation is that the sex and treatment effects in HVC reflect increases in mature BNDF, and not proBDNF, in males and E2-treated females. At the age our data were collected (post-hatching day 25), males already have more cells in HVC than females [62]. Thus, the sex difference we detected by immunohistochemistry could simply reflect a difference in cell survival or addition caused by other factors. The sex-specific E2 effect is consistent with the idea that BDNF up-regulation may be one mechanism by which E2 increases cell survival in masculinization of the female HVC. Alternatively, it is possible that a general increase in HVC cell number due to E2 treatment of females (*e.g.*, [20]) is regulated by an independent mechanism, and the enhanced expression of BDNF that we detected simply reflects the survival of these cells. Future studies should address these ideas.

E2's increase of TrkB-FL in HVC across the two sexes provides an opportunity for the function of BDNF to be enhanced, but also suggests that the mechanism underlying the female-specific increase in BDNF+ cells by E2 is not regulated by differential availability of this receptor. In contrast, the greater expression of TrkB-T (which facilitates apoptosis, see above), in females compared to males could generally serve to facilitate the development of sex differences in cell number in HVC.

Unlike HVC, in RA the results on BDNF from Western blot analysis and immunohistochemistry were parallel. The fact that relative proBDNF concentration and the estimated total number of cells expressing BDNF were both greater in males than females, and that E2 increased both measures in only females, suggest that what was detected by immunoshistochemistry may have largely been proBDNF. Alternatively, the pattern of mature BDNF expression across groups may mirror that of proBDNF. Further work is needed to distinguish between these possibilities. Regardless, the fact that overall cell number in RA does not diverge between the sexes until after day 25 [62] suggests that sex differences in BDNF labeling by immunohistochemistry are not the passive result of the cell loss that begins to occur around this time. In contrast, greater expression of BDNF might actively promote greater survival of cells in the RA of males compared to females, and E2 might masculinize this function in females.

In addition to directly testing these hypotheses, it will be important to determine why E2 affected BDNF in only females both in RA and HVC (see above). One possibility is that males' responses to endogenous E2 may have already been maximized so no further change due to exogenous E2 was exhibited. Another possibility is that estrogenic up-regulation of BDNF is modulated by activity of one or more sex chromosome genes. Both Z and W genes exhibit differential expression between the sexes, including the brain [32], [34], [35], [36], [63], so numerous candidates are plausible.

As noted in the Introduction, TrkB is on the Z-chromosome, and its mRNA is increased in developing males compared to females, at least in HVC. However, the present data do not support the idea that either form of the TrkB protein is a key contributor to masculinization in HVC or RA. In RA, only TrkB-T could be quantified, and expression of this protein was not sexually dimorphic. In HVC, the relative concentration of TrkB-FL was equivalent between the sexes, and TrkB-T was increased in females. Thus, while this truncated isoform could be critical for feminization (or demasculinization), it is an unlikely candidate for enhancing structure or function of HVC. This truncated isoform largely inhibits BDNF functions such as cell survival, and complementarily BDNF negatively regulates TrkB-T mediated cytoskeletal changes, such as dendritic growth [11].

In LMAN, the number of BDNF+ cells was greater in females than males at post-hatching day 25. Unlike other song control nuclei, LMAN exhibits similar volume and dendritic morphology between the sexes at this age. Soma size is also equivalent in males and females until at least day 50 [64]. Thus, while changes in cell number across development of LMAN are not completely clear [64], available information is consistent with the idea that this variable is also equivalent between the sexes at this juvenile stage. It is not obvious whether the larger BDNF+ cell number in females reflects the pro- or mature form, or a combination of both. However, the fact that proBDNF concentration as detected in Western blot analysis did not differ between the sexes whereas immunohistochemically labeled cells did, suggests that this labeling may largely reflect the mature peptide. It is also possible that while females have more proBDNF+ cells they express less per cell, resulting in a lower concentration of proBDNF detected by Western blot analyses.

If the mature BDNF is in fact greater in females than males, one intriguing possibility is that this female-biased effect, which does not occur in the other song nuclei analyzed, is what keeps LMAN morphology at this age similar between the two sexes (for example, see [65]). If factors, perhaps including those encoded on sex chromosomes, generally induce differentiation of neural structure and function, then a larger number of BDNF cells in the LMAN of females might prevent demasculinization that would have otherwise occurred. Such a process may be particularly important at post-hatching day 25, because at this age both males and females appear to learn characteristics of adult song [66], and LMAN may play a critical role [64].

In this brain region, E2 increased the relative concentration of proBDNF but decreased the estimated total number of BDNF+ cells. These results are difficult to interpret both because of their opposite directions and because little is known about the function of E2 in LMAN development. While just speculation, this pattern is consistent with the possibility that E2 increases BDNF synthesis while also facilitating release of the mature peptide, which could limit detectability by immunhistochemistry. The studies examining the masculinizing effects of this hormone have not quantified features of LMAN morphology or its connections [67], [68], [69]. However, as early E2 can masculinize song learning [70], [71], perhaps effects mediated by forms of BDNF are more behavioral than structural. That TrkB-T exhibited no obvious effect of sex or treatment is consistent with the fact that this brain region will after day 30 shrink in parallel in the two sexes [64].

Finally, in Area X (or the equivalent portion of the MSt in females), Western blot analyses did not reveal effects of sex or treatment on either proBDNF or TrkB-T. However, the fact that BDNF+ cell number was equivalent across control males and E2-treated individuals of both sexes indicates that E2 in fact masculinized the number of cells expressing BDNF peptides in females. This effect is consistent with an E2- induced appearance of a distinct Area X with Nissl-staining [20], [21], [46]. We do not have sufficient information to know whether BDNF is a component of the process through which this morphology is masculinized or whether up-regulation of this peptide is a consequence of the masculinization. However, the absence of effects on both proBDNF and TrkB-T suggests that potential for mature BDNF to be involved in masculinization of Area X. As suggested above, it is possible that males are also normally affected by E2, but exogenous treatment produced no further response above that of the endogenous effect.

### Future Directions

The present data provide some novel information regarding expression patterns of specific isoforms of BDNF and TrkB. Considered in the context of prior work on these molecules and the genes that encode them (see above), it is clear that BDNF and perhaps ligand-independent actions of TrkB-T could play key roles in shaping development of song system structure and/or function. The challenge now is to more fully describe developmental changes in pro- and mature forms of BDNF and the full-length and truncated forms of TrkB to see how they parallel known changes in morphology and song learning. Then, manipulations of availability and activity can begin to elucidate the specific functional roles of each peptide and how they may interact with E2 to induce sexual differentiation.
